# Predicting speech discrimination scores from pure-tone thresholds—A machine learning-based approach using data from 12,697 subjects

**DOI:** 10.1371/journal.pone.0261433

**Published:** 2021-12-31

**Authors:** Hantai Kim, JaeYeon Park, Yun-Hoon Choung, Jeong Hun Jang, JeongGil Ko

**Affiliations:** 1 Ajou University Hospital, Suwon, South Korea; 2 Department of Otolaryngology, School of Medicine, Ajou University, Suwon, South Korea; 3 School of Integrated Technology, College of Engineering, Yonsei University, Seoul, South Korea; National University of Sciences and Technology, PAKISTAN

## Abstract

Diagnostic tests for hearing impairment not only determines the presence (or absence) of hearing loss, but also evaluates its degree and type, and provides physicians with essential data for future treatment and rehabilitation. Therefore, accurately measuring hearing loss conditions is very important for proper patient understanding and treatment. In current-day practice, to quantify the level of hearing loss, physicians exploit specialized test scores such as the pure-tone audiometry (PTA) thresholds and speech discrimination scores (SDS) as quantitative metrics in examining a patient’s auditory function. However, given that these metrics can be easily affected by various human factors, which includes intentional (or accidental) patient intervention, there are needs to cross validate the accuracy of each metric. By understanding a “normal” relationship between the SDS and PTA, physicians can reveal the need for re-testing, additional testing in different dimensions, and also potential malingering cases. For this purpose, in this work, we propose a prediction model for estimating the SDS of a patient by using PTA thresholds via a Random Forest-based machine learning approach to overcome the limitations of the conventional statistical (or even manual) methods. For designing and evaluating the Random Forest-based prediction model, we collected a large-scale dataset from 12,697 subjects, and report a SDS level prediction accuracy of 95.05% and 96.64% for the left and right ears, respectively. We also present comparisons with other widely-used machine learning algorithms (e.g., Support Vector Machine, Multi-layer Perceptron) to show the effectiveness of our proposed Random Forest-based approach. Results obtained from this study provides implications and potential feasibility in providing a practically-applicable screening tool for identifying patient-intended malingering in hearing loss-related tests.

## 1 Introduction

Pure-tone audiometry (PTA) tests and speech discrimination scores (SDS) are commonly used for examining a patient’s auditory function in today’s clinical practice. PTA quantifies the hearing level for each audible frequency, by asking patients to listen to a pure tone in a soundproof facility, with the test sequence designed to observe the response to tones with varying frequencies from the test subject.

**Fig 1:**
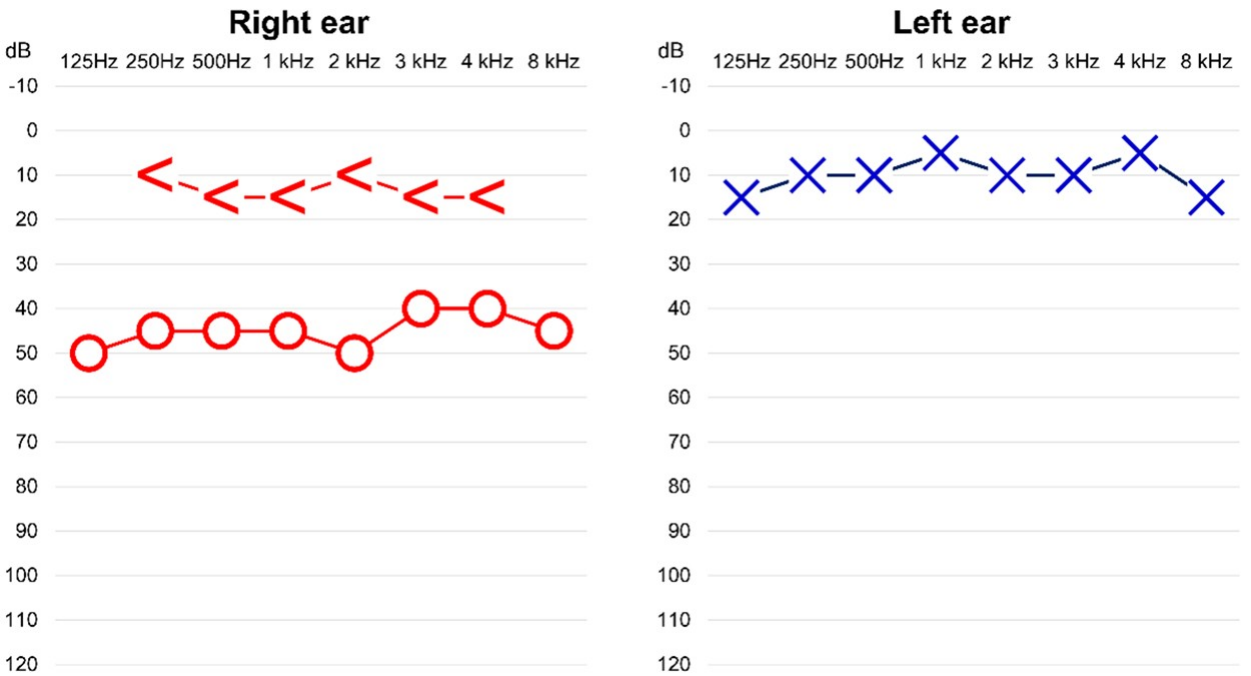
A sample audiogram from PTA. Left ear presents normal function of hearing. However, in right ear, there is a difference between air conductive (i.e., arrow) and bone conductive thresholds (i.e., circle). The audiogram for this patient suggests that there may be clinical issues related to the middle or external ear.

Specifically, PTA targets to determine and quantify the degree of hearing loss by measuring the hearing conditions in two different ways: 1) air conductive and 2) bone conductive hearing levels. The goal of the air conduction measurements is to diagnose the current hearing level irrespective of the external, middle, and inner ear conditions, and the bone conduction hearing levels offer focused hints on the functional status of the inner ear. In current day clinical practice, a person’s hearing level is described as a set of thresholds, in which the minimum audible threshold level is taken as the patient’s hearing level. For example, if a patient responds to a pure tone with 40 dB loudness at a specific frequency, but does not at 35 dB for the same frequency, the patient’s hearing threshold (for that frequency) is defined as 40 dB. Intuitively, the lower the threshold, the better the hearing function of the patient. The PTA thresholds are measured at different frequencies (e.g., 125, 250, 500, 750, 1000, 1500, 2000, 3000, 4000, and 8000 Hz; note: 3000 and 8000 Hz are not used for bone conduction tests), and as the example in Fig 1 shows, the final results from this test is summarized in the form of an audiogram for each ear. Such audiograms serve as the most fundamental core in evaluating hearing functions of a person and when diagnosing otologic diseases [1].

While being widely used due to its simple testing protocol, unfortunately, PTA is limited to only measuring binary measurements (i.e., can hear or cannot hear a frequency tone at target level) and does not qualitatively measure the ability of a person to recognize speech; thus, it is insufficient in comprehensively examining a patient’s auditory function as a whole. To supplement such limitations, three other types of speech tests results are commonly measured: the speech-detection threshold (SDT), the speech-reception threshold (SRT), and the speech discrimination score (SDS). Of these three tests, SRT and SDT measure audible thresholds, similar to PTA, using non-pure tone, monophonic or disyllabic words as test modality. SRT and SDT are also being widely used but they are limited as secondary tools to assess audible thresholds. On the other hand, SDS is widely considered to be clinically important [2]. It not only evaluates a patient’s communication ability, but also plays an essential role in determining the method of treatment for addressing the patient’s hearing loss [1, 3, 4]. To measure the SDS of a patient, the patient listens to and repeats monosyllable words spoken by the examiner; the correct answer rate (in %) is output as the final score for the patient. A typically used monosyllable words list consists of 25 or 50 phonetically balanced (PB) words in the person’s native language, in our case, Korean. When hearing capabilities are considered normal, PB words are typically uttered at ∼40 dB loudness, while words can be given at the most comfortable loudness (MCL) level for patients with hearing loss [1].

PTA and SDS tests have different objectives in the audiologic test suite, however, the correlation between these two test results is not surprising. In fact, the two tests are, in many cases, considered to complement each other [5–7]. Such complementary correlation is usually helpful in clinical diagnosis to conduct cross-validation on test results captured from various dimensions and perspectives. For example, by analyzing the two results comprehensively, we can diagnose a patient with pathological retrocochlear lesion who would typically show a low SDS even with close-to-normal pure-tone thresholds [8]. Furthermore, the correlation between the PTA thresholds and SDS can help cross-evaluate the reliability of the examination process itself. If the SDS is measured to be noticeably low (or high) compared to the patient’s corresponding pure-tone threshold measurements, an otolaryngologist can suspect weak reliability and suggest additional in-depth examinations.

Mismatch events often occur given that both PTA and SDS tests rely mostly on patients’ subjective responses, in other words, patients can easily intervene the tests with intentional negative (or improper) responses toward the stimuli. There can be many reasons behind such behavior, which in some cases is closely tied with monetary benefits issued from the government of insurance companies, when hearing loss is officially diagnosed. For this, experienced otolaryngologists and audiologists will suspect patient-initiated malingering to some extent by reviewing and comparing consequences of the two tests. However, there is no rigid criteria for determining the correlation between PTA and SDS. In practice, there can be alternative audiologic tests, such as auditory brainstem response (ABR) and auditory steady-state response (ASSR), which do not exploit patients’ subjective responses. However, such objective evaluations are expensive and are of additional burden for otolaryngologists when performed frequently. Furthermore, these measurements do not possess as much clinical value as PTA and SDS under the premise that all tests were properly performed. As such, malingering activity detection is one potential application that can be enabled when the PTA-SDS relationship of a large population is known to physicians. This knowledge can also be generically applied to various clinical decision support by allowing the physicians to easily identify samples that need careful considerations with minimal manual effort in filtering them.

The core hypothesis we make in this work is that by computing a methodological relationship between PTA and SDS, we can potentially resolve the aforementioned issues. In fact, there have been a number of previous efforts to predict an expected SDS from PTA test results. For example, Yoshioka and Thornton designed a method for predicting SDS from PTA thresholds collected from 529 ears [9]. However, the performance of the proposed model was limited to an *R*2 score of only 0.58-0.60. Marshall and Bacon also proposed a formula to predict SDS using PTA thresholds (at 2 kHz) and the patient’s age using stepwise multiple regression [10]. Unfortunately, this study also reported an unsatisfactory correlation coefficient between the predicted SDS and the actual SDS of 0.67, which is considered to be too low to be considered generally acceptable. While such conventional statistical method-based approaches are still meaningful efforts, the low prediction accuracy limited their use in practical clinical protocols.

More recently, with breakthroughs in machine learning algorithms and the increased accessibility to various forms of healthcare and clinical data, more intelligent algorithms have been introduced to clinical applications and are being applied to various domains [11–18]. In this work, we follow the paradigm of exploiting machine learning algorithms together with clinical data and compare candidate models that can suit our purposes. Specifically, we evaluate the performance of widely used models such as Support Vector Machines (SVMs), Multi-layer Perceptrons (MLPs), and Random Forest Models. Note that the features that need to be used from the input data is clear; thus, a machine learning approach is sufficient, and a more complex deep learning approach is not suitable in our application. Using the three machine learning approaches, we present performance comparisons in predicting SDS using PTA thresholds as input to show that such machine learning-based schemes can overcome the accuracy limitations of conventional statistical methods used in previous work.

A major hurdle in applying machine learning to such an application is the need for a much larger quantity dataset compared to traditional statistical methods. For this reason, we collect and exploit data from 12,697 subjects who underwent both the PTA and the speech discrimination tests. We use this data to train and evaluate the three different machine learning-based approaches. Our evaluations on these three potential solutions show that the robustness nature of the Random Forest model allows for a high prediction accuracy (with cross validation) of 96.64%, which outperforms those reported from previous studies based on statistical models. The high accuracy achieved by our scheme suggests that a machine learning-based approach can be effective enough to be applied in real-world clinical practice.

## 2 Methods

The data used in our work is a large-scale dataset of PTA and SDS scores collected from 12,697 subjects at the Ajou University Hospital, a large-sized general hospital located in Suwon, South Korea. The subjects present an average age of 49.1 ± 18.8 (min 3; max 101), with 48.3% being male (6,132 subjects) and 51.7% female (6,565 subjects). PTA was performed as part of typical medical examinations (e.g., checkups) or for diagnosing otologic diseases such as middle or external ear abnormality or sensorineural hearing loss. For the examination, pure tone stimuli were given at 250, 500, 1000, 2000, 3000, 4000, and 8000 Hz for air conductive measurements and 250, 500, 1000, 2000, and 4000 Hz was used for the bone conductive measurements. The air conductive and bone conductive PTA thresholds, were taken separately for each ear, right and left, of the subjects at the aforementioned frequencies. For speech discrimination score collection, subjects were given 25 monosyllable PB words at the most comfortable loudness level (MCL) measured in the PTA test. Usually, subjects with normal hearing conditions were given words at around 40 dB and the MCL level was used for subjects with hearing loss. Specifically, the examiner called out a total of 25 monosyllable words in the magnitude of MCL, and the examinee followed by repeating the words. In this process, 4 points were given for each word that the examinee correctly repeated, resulting in a total score of 100 points.

Using this data, we designed three machine learning-based SDS prediction models using 14 features captured from the air conductive PTA (AC PTA) tests and 10 features from the bone conductive PTA (BC PTA) tests (i.e., PTA thresholds for each tested frequency at each ear). Specifically, we select the Support Vector Machine, Multi-layer Perceptron, and Random Forest machine learning models as potential approaches. All 24 features were used to predict the SDS of a target subject (12 features for each ear), which is a score given on a scale of 0–100. We note once more that the PTA features and ground-truth SDS data are correlated on a per-ear-basis, where the PTA samples from the right ear were used to predict the right ear SDS, and the PTA samples from the left ear were used for left ear SDS prediction. All data collection and processing research presented in this work was approved by the Institutional Review Board at Ajou University Hospital (AJIRB-MED-MDB-19-344).

### 2.1 Data preprocessing

As Table 1 shows, given an SDS score range of 0–100, we first created 10 bins each with size 10 (with exception to the final bin which ranged from 90–100 with size 11). The goal of our SDS prediction system was to classify which SDS score bin the PTA-based features of a subject would most likely belong to, as small differences in SDS would not affect clinical decisions (e.g., the clinical outcome differences between PTA of 51 and 59 is not significant [19, 20]). Unfortunately, deeper observation on the collected data revealed that the SDS of the samples from 12,697 were not uniformly distributed over the 10 bins. In particular, as we show in Table 1, 60.9% of the samples (7,737 among 12,697) were classified in the final bin (i.e., bin covering 90–100 SDS), and only 0.5% of the samples (75 among 12,697) belonged to the first (i.e., 0–10 SDS). Such an unbalanced data set suggests that supervised machine learning model architectures may not be trained properly due to the small number of samples available in less frequently observed categories. Specifically, given such an imbalanced dataset, there is a high chance that the prediction results will be biased given that more training opportunities are available for the classes with more input data. One possible approach to address such data imbalance is to “undersample” by removing samples from categories with large quantities, but with the first bin having only 75 samples, such an approach would result in eliminating too many data samples from the training set and limit the classification performance [21]. In fact, our preliminary evaluations using the undersampling approach showed significantly low performance due to fuzzified bin boundaries caused from high variances from the lack of samples.

**Table 1:** The table of SDS bin distribution on 12,697 subjects.

SDS bin	The number of subjects for ‘left’ ear SDS (%)	The number of subjects for ‘right’ ear SDS (%)
0–9	75 (0.59)	301 (2.37)
10–19	111 (0.87)	181 (1.42)
20–29	299 (2.35)	328 (2.58)
30–39	221 (1.74)	261 (2.05)
40–49	422 (3.32)	396 (3.11)
50–59	340 (2.67)	313 (2.46)
60–69	719 (5.66)	702 (5.52)
70–79	721 (5.67)	624 (4.91)
80–89	2052 (16.16)	1848 (14.55)
90–100	7737 (60.93)	7743 (60.98)

Therefore, in this work we adopted an “oversampling-based approach”, which is a well-known technique to improve the classification performance for imbalanced datasets [22]. Specifically, for training purposes, we made replicated samples from classes with low sample-counts to match those of larger sample-count categories. We select the 80% sample count of the samples from the largest bin to be the target oversampling count for all bins (with 7,737 and 7,743 being the largest bin count for the left and right ears, respectively, we set the oversampling target to 6,189 and 6,194 for the left and right ears). Among the samples in each bin, we take 80% of the samples and replicate them multiple times to match the target oversampling count. We choose the 80% threshold given that, as we discuss later, we target to validate the proposed scheme using 5-fold cross validation. In other words, we do so to completely separate the training samples (as part of the oversampled elements) from the test dataset. Note that such a simple approach is very powerful in balancing the dataset, and has also been applied in a number of previous work [23–25]. Compared to the downsampling approach, oversampling allows the model to maintain an exploit the full distribution/complexity characteristics of the original dataset.

We emphasize once more that in this oversampling process we made sure that the samples used for training and verification/testing were clearly separated, so that oversampling was performed for only the training set data. One important point is that data oversampling can potentially lead to the model overfitting itself to the training set; resulting in a model that is only accurate for the trained dataset. Thus, we needed to take a preventive approach so that the SDS prediction model could be generalized to data collected from larger populations. As we later discuss, among different machine learning models, this was one of the core reasons that the Random Forest model performs the best, as it is architecturally known to be robust against model overfitting [26].

### 2.2 Machine learning model design

#### 2.2.1 Support vector machine-based model

The Support Vector Machine (SVM) is one of the most commonly used machine learning model, which essentially targets to define classification boundary for a given dataset of multiple classes. In a number of previous work, SVMs have shown to be very powerful in classifying both binary and multiple classes in a given dataset [27, 28]. SVMs allow for the configuration of different kernel functions based on the characteristics of the target datasets. In our case, given the non-linearity of the dataset features, we exploit the radial basis function (RBF) kernel in our SVM implementations. We also set 10 C as the regularization parameter, and 0.001 gamma for the RBF kernel’s coefficient.

#### 2.2.2 Multi-layer perceptron-based model

The Multi-Layer Perceptron (MLP) model is a widely used machine learning model in the form of a feed-forward deep neural network. Specifically, MLP models consist of a number of layers containing a network of neurons, which are connected with different weights. The weights are trained (and identified) in the model training phase, making the MLP model suitalble for complex data relationships that show non-linear patterns. MLP models are theoretically known to be capable of fitting a wide range of smooth, non-linear functions with high accuracy [29, 30]. We configure 50, 100, and 150 hidden layers in our MLP model with ReLu as the activation function, and apply the Adam optimizer. Other hyperparameters for this model were configured to the default values on the Scikit-learn framework.

#### 2.2.3 Random Forest-based model

The Random Forest model is a widely-used model known to offer high accuracy with minimal computational complexity [31–33]. Specifically, the Random Forest model is fundamentally an ensemble model which consists of multiple decision trees and passes new data simultaneously through each tree architecture. The “forest” of trees then votes based on the results obtained from each decision tree to select the decision with the most votes as its final classification decision. Such an ensemble-based approach is the reason behind the model’s robustness towards the overfitting issue. Another important feature of the Random Forest model to emphasize is its “bagging” feature. Bagging, which is a widely used term for bootstrap aggregation, allows each tree in the Random Forest to randomly select inputs from a large set of input values. This again is an important operation that allows the Random Forest model to tolerate high levels of input noise and imbalanced datasets [26, 32].

Particularly, for the Random Forest model, we configure 1,000 tree-based estimators (i.e., number of trees) to construct a forest and exploited the Gini Impurity [34, 35] as the criteria to measure split quality. Note that in Random Forest models, a “split” takes place when a tree branches out, and the quality of a split is measured to assure high quality decision trees within the forest. Other hyperparameters for model configurations were set to the default values on the scikit-learn framework we used for the implementation [36]. Detailed parameters for our Random Forest model can be found in Table 2.

**Table 2:** The hyper-parameters used in our proposed Random Forest model provided by scikit-learn framework.

Hyper-parameter	Value	Description
n_estimator	1000	The number of trees in the forest.
criterion	gini	The function to measure the quality of a split.
max_depth	None (default)	If ‘None’, then nodes are expanded until all leaves are pure (i.e., single sample) or until all leaves contain less than ‘min_samples_split’ samples.
min_samples_split	2 (default)	The minimum number of samples required to split an internal node.
min_samples_leaf	1 (default)	The minimum number of samples required to be at a leaf node.
max_features	auto (default)	The number of features for the best split. If ‘auto’, then ‘max_features’ is the square root of ‘n_features’ (i.e., the number of features).
max_leaf_nodes	None (default)	The maximum number of leaf nodes. If ‘None’, then unlimited number of leaf nodes.

## 3 Classification results

For evaluations, we performed a 5-fold cross-validation over the data collected from 12,697 patients. In all five runs 80% of the data was selected to be the training set and the remaining 20% was used for testing the machine learning models. As mentioned earlier, we performed oversampling only for the training samples and the test samples were left unaltered. For each test run, a different set of test and training data was selected to assure that all samples participate in the test dataset once over all five test runs.

### 3.1 Dataset

Our machine learning models are designed so that it makes accurate predictions on the left and right ear SDS from the PTA data collected from 12,697 subjects. As briefly mentioned earlier, we collected AC pure-tone thresholds measured from both ears at frequencies of 250, 500, 1000, 2000, 3000, 4000, and 8000 Hz, and collected BC pure-tone thresholds at 250, 500, 1000, 2000, and 4000 Hz. The average of the thresholds was extracted using the four-frequency (i.e., 0.5, 1, 2, and 4 kHz) method [1], which is a conventionally used average hearing computation process in clinical practice. Corresponding ground truth SDS measurements were measured at 40 dB or at the MCL, depending on the subject’s hearing abilities, with 25 PB monosyllable Korean words. We split this dataset into PTA and SDS datasets, and trained the models for each ear, respectively.

### 3.2 Machine learning model comparisons

**Fig 2:**
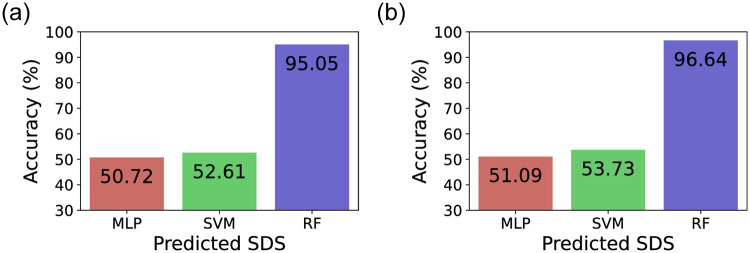
SDS prediction results using three models, multilayer perceptron, support vector machine, and Random Forest. For training and testing of the three models, a dataset divided into 10 bins was used for training and evaluation. (a) Left ear, (b) Right ear.

In Fig 2 we present the overall SDS classification results using PTA threshold data for the three different machine learning models evaluated in this study. Specifically, we present the classification accuracy for the left and right ears, respectively. As the plots show, the classification results using the Random Forest-based approach shows noticeably higher performance compared to the SVM and MLP-based approaches. This is mainly due to the fact that the hyperparameter optimization can be extremely challenging sensitive and can heavily affect the performance for SVM and MLP models. Despite selecting the best possible parameters for a target input dataset, as we perform multiple folds to cross validate the generality of the model. On the other hand, Random Forest models are designed to reduce the variability of predictions across datasets and minimizes the chances of overfitting. Such a phenomena of Random Forest models out performing SVM and MLP models is not always true for all data types, but has been observed in a number of previous work as well [17, 37].

These results motivate us to select the best possible machine learning approach suitable for our dataset, and based on the results in Fig 2 for the remainder of this work, we select the Random Forest model-based approach as our core approach and present detailed evaluations using this configuration.

### 3.3 SDS prediction

**Fig 3:**
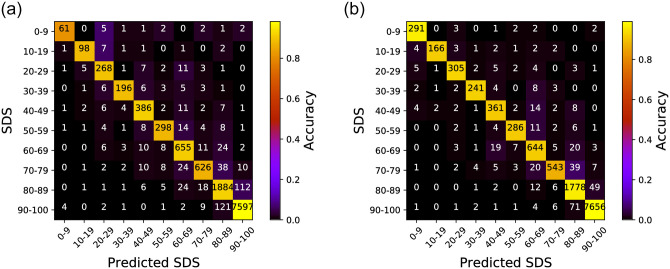
Confusion matrix for the SDS prediction performance using PTA thresholds. The X-axis shows the predicted SDS and the Y-axis shows the ground-truth. The numbers in the matrix present the number of prediction occurrences. An ideal confusion matrix would have bright colors (high occurrence counts) diagonally. (a) Left ear, (b) Right ear.

We now take a deeper look into the results from the Random Forest-based model and start by observing the confusion matrix for SDS prediction. As mentioned, the overall SDS classification accuracy of 95.05% and 96.64% for the left and right ears, respectively. We present the confusion matrices for the two cases in Fig 3 and detailed performance results in Table 3. Comprehensively these results re-confirm that our proposed Random Forest model shows very accurate SDS prediction performance with PTA threshold inputs. More specifically, the precision, computed by dividing the number of true positive cases with the sum of false positive and true positive cases (i.e., a prediction is true if the prediction is correct and false otherwise), was 90.64% for the left ear and 94.11% for the right. The recall, which takes the true positive cases and divides this with the sum of true positive and false negative counts, was 89.49% and 93.00% for the left and right ears, respectively. Lastly, in terms of the F1-score, which is the harmonic average of the precision and recall, the left and right ears showed 90.03% and 93.52%, respectively. We later present detailed discussions on some of the interesting points identified from these results, but overall, we can see that our proposed model shows superior performance compared to previously proposed SDS prediction schemes.

**Table 3:** Accuracy, precision, recall and F1 score of our SDS prediction model for the right and left ears.

SDS prediction	Left ear (%)	Right ear (%)
Accuracy	95.05	96.64
Precision	90.64	94.11
Recall	89.49	93.00
F1 score	90.03	93.52

While we show that the proposed scheme’s SDS prediction is satisfactory, we statistically analyze the prediction performance using a Wilcoxon signed rank-based statistical analysis [38]. Prior to this, we confirm the normality of the ground truth SDS and predicted SDS scores through the Shapiro-Wilk test [39]. The statistical *w*, which represents the test statistics, is 0.60 for both SDS score sets, and the p-value between the two sets is < 0.001. We also note that with Levene’s homoskedasticity test [40], the p-value is 0.94, suggesting that the dataset shows normality. Finally, via the Wilcoxon signed rank test on the two sets, we observed a p-value of 0.69, which rejected the null hypothesis (*H*_0_). Thus, we can claim that there is statistically minimal difference (*H*_1_) between the ground truth SDS and predicted SDS scores.

### 3.4 Age group prediction

Age-related hearing loss takes place from the middle ages and on-wards. While most people will experience gradual change in speech recognition, sudden changes of hearing at earlier ages is considered unnatural. Factors such as being involved in accidents or hazardous environments can cause abnormal hearing loss. Therefore, subjects with no special concern will show similar PTA and SDS measurement “trends” when being part of a similar age group. This also suggests that by making age group predictions using PTA measurements (or SDS), and comparing the predicted age with the actual age of the subject can serve as an easy-to-access indicator on whether the measured values fall in the “normal range”, or deeper investigation is required.

For this purpose, we evaluated how well a Random Forest-based machine learning model could predict the subject’s age group using PTA features as the model input. Note that for age group estimation, we configured the parameters of the Random Forest model and the contents in the PTA training dataset to be identical to the previous experiment (for SDS prediction). The only difference here is that, for age group prediction, we utilized the subjects’ age (with bin sizes of 10 as in Table 4) as the ground-truth data in the training phase. As aforementioned, the 12,697 subjects had an average age of 49.1 ± 18.8; thus, we exploited a dataset covering a wide range of age groups.

**Table 4:** The table of age distribution on 12,697 subjects.

Age bin	The number of subjects (%)
0–9	303 (2.38)
10–19	957 (7.53)
20–29	868 (6.83)
30–39	1383 (10.89)
40–49	2255 (17.76)
50–59	3032 (23.88)
60–69	2122 (16.71)
70–79	1400 (11.02)
80–89	359 (2.82)
90–100	17 (0.13)

**Fig 4:**
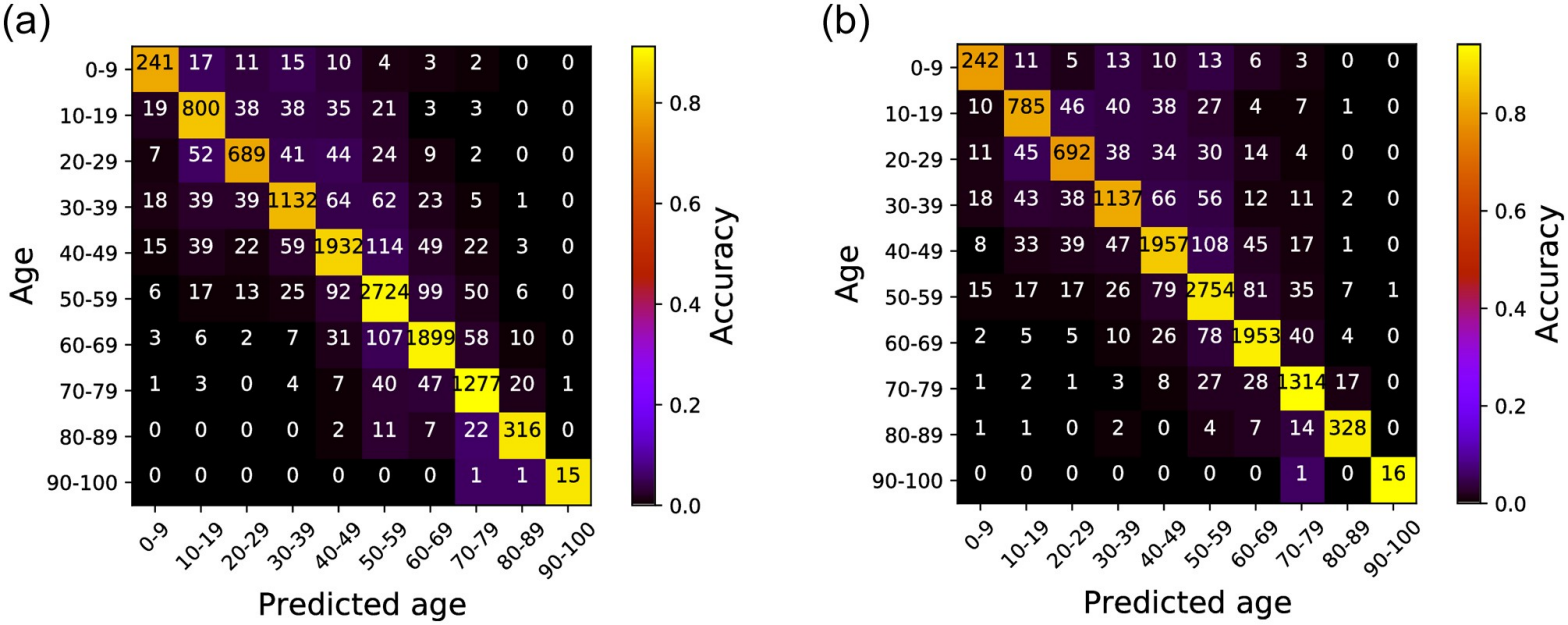
Confusion matrix for the age group prediction performance using PTA thresholds. The X-axis shows the predicted age group of the patient and the Y-axis shows the ground-truth. The numbers in the matrix present the number of prediction occurrences. An ideal confusion matrix would have bright colors (high occurrence counts) diagonally. (a) Left ear, (b) Right ear.

The confusion matrix results in Fig 4 presents a visual representation of the age group prediction performance of our proposed scheme. Overall, quantitatively, as Table 5 shows, the average age prediction accuracy showed 86.83% for the left ear and 88.03% for the right. Specifically, the Random Forest model showed 78.58% and 87.44% precision for the left and right ears, and 77.91% and 87.31% recall for each ear. Lastly, the F1-scores for the two ears showed 78.22% and 87.36%, respectively. Again, this indicates that the PTA scores can potentially be a good indicator for the expected age group of a subject, which can be an important clinical indicator for detecting abnormal hearing loss conditions of a patient.

**Table 5:** Accuracy, precision, recall and F1 score of age prediction for the right and left ears.

Age prediction	Left ear (%)	Right ear (%)
Accuracy	86.83	88.03
Precision	78.58	87.44
Recall	77.91	87.31
F1 score	78.22	87.36

### 3.5 Characteristics of subjects with large SDS estimation errors

Finally, we performed deeper analysis into the characteristics of patients that showed a large difference between the actual SDS (i.e., ground-truth) and predicted SDS (Table 6). Specifically, we present cases in which the predicted SDS bin was 6 bins (or more) away from the measured SDS score bin. Surprisingly to note, the many predictions of patients with the largest difference were caused from human errors in the recording phase. Note that all of these measurements took place manually (following common clinical protocols) by audiologists; thus, a level of human error is expected in such large datasets. Note that PTA and speech discrimination examinations are performed using the audiometer device. Typical audiometers have the capability of autonomously transmitting the information to the patients’ EMR. However, the device that we used for our data collection phase was not compatible with the hospital’s EMR and needed the values to be manually transferred. Errors in measurements are typically captured when clinicians perform diagnosis, but due to regulatory issues, they are not removed from the EMR (despite being faulty). When gathering our dataset from the EMR, this information was not labeled and included in the dataset. We also noticed that subjects with head trauma history showed rapid hearing loss compared to others in similar age groups, causing prediction errors in both SDS and age group.

**Table 6:** Clinical remarks for subjects showing large bin differences (≥ 6) between predicted SDS and actual SDS.

Left ear SDS Results			Right ear SDS Results		
Patient (gender / age)	Bin distance gap	Clinical remarks	Patient (gender / age)	Bin distance gap	Clinical remarks
F/20	9	Incorrectly recorded (Error)	M/21	9	Incorrectly recorded (Error)
M/27	9	Incorrectly recorded (Error)	M/19	9	Incorrectly recorded (Error)
F/58	9	Incorrectly recorded (Error)	F/47	7	Incorrectly recorded (Error)
M/36	9	Incorrectly recorded (Error)	F/66	7	Progressive hearing loss (for 35 years) from young age
M/52	9	Incorrectly recorded (Error)	M/64	7	Hearing loss after severe head trauma
M/16	9	Incorrectly recorded (Error)	M/40	6	Hearing loss after accident
F/81	7	Age-related hearing loss	M/53	6	Sudden sensorineural hearing loss
M/29	7	Progressive hearing loss of unknown cause	M/11	6	Progressive hearing loss of unknown cause
F/59	7	Hearing loss after head trauma	M/78	6	Age-related hearing loss
M/64	7	Conductive hearing loss by chronic otitis media	M/54	6	Sudden sensorineural hearing loss
F/65	7	Head injury by domestic violence	F/80	6	Age-related hearing loss
F/34	7	Progressive hearing loss of unknown cause	F/23	6	Hearing loss after acoustic trauma
F/78	7	Age-related hearing loss	F/60	6	Meniere’s disease
F/63	6	Meniere’s disease	F/52	6	Otitis media with effusion
F/28	6	Progressive hearing loss of unknown cause	F/29	6	Progressive hearing loss of unknown cause
F/36	6	Progressive hearing loss of unknown cause			
F/47	6	Progressive hearing loss of unknown cause			

## 4 Discussion

### 4.1 Prediction on age

While our proposed scheme well-predicts the patient age groups, one interesting point to notice from the results in Fig 4 is that for both ears, the prediction performance is better for the elder population when compared to the age groups between 0–49. This is because age-related hearing loss is unusual in this younger age groups. Patients aging 0-49 years mostly take hearing tests for examining various otologic diseases, but not for age-related hearing loss. Nevertheless, achieving high age prediction accuracy in this group can still be useful in clinical practice. As an example, one of the difficulties in evaluating industrial accident compensations for noise-induced hearing loss is that the patient’s hearing loss is a mixture of the noise-induced and senile hearing loss. If there is a large difference between the age predicted by the patient’s PTA and SDS via our scheme and the actual age, it is likely that hearing loss has progressed by unnatural factors other than aging.

### 4.2 Difference in the SDS prediction between left and right ears

We now discuss an interesting observation from the classification result for the 80-90 and 90-100 bins in Fig 3. First, we look deeper into why the left ear in Fig 3(a) shows more misclassified cases (nearly twice) than the right ear in Fig 3(b). Specifically, while most cases in Fig 3 are well-classified with high accuracy, we can notice some parts of the confusion matrix in which a significant number of misclassified cases exist, but with different patterns for the left and right ears. As an example, focus on the results for the 80-90 and 90-100 bin regions on the confusion matrix. Here, for the left ear (c.f., Fig 3(a)), our model *incorrectly* predicts a total of 112 samples with SDS 80-89 as the ground-truth to be SDS range 90-100. In contrast, for the right ear presented in Fig 3(b), the same misclassification occurs only for 49 samples, which is only half of the left ear case. Such similar trends can also be seen for SDS 90-100 ground-truth cases misclassified as SDS range 80-89 (i.e., 121 vs 71).

**Fig 5:**
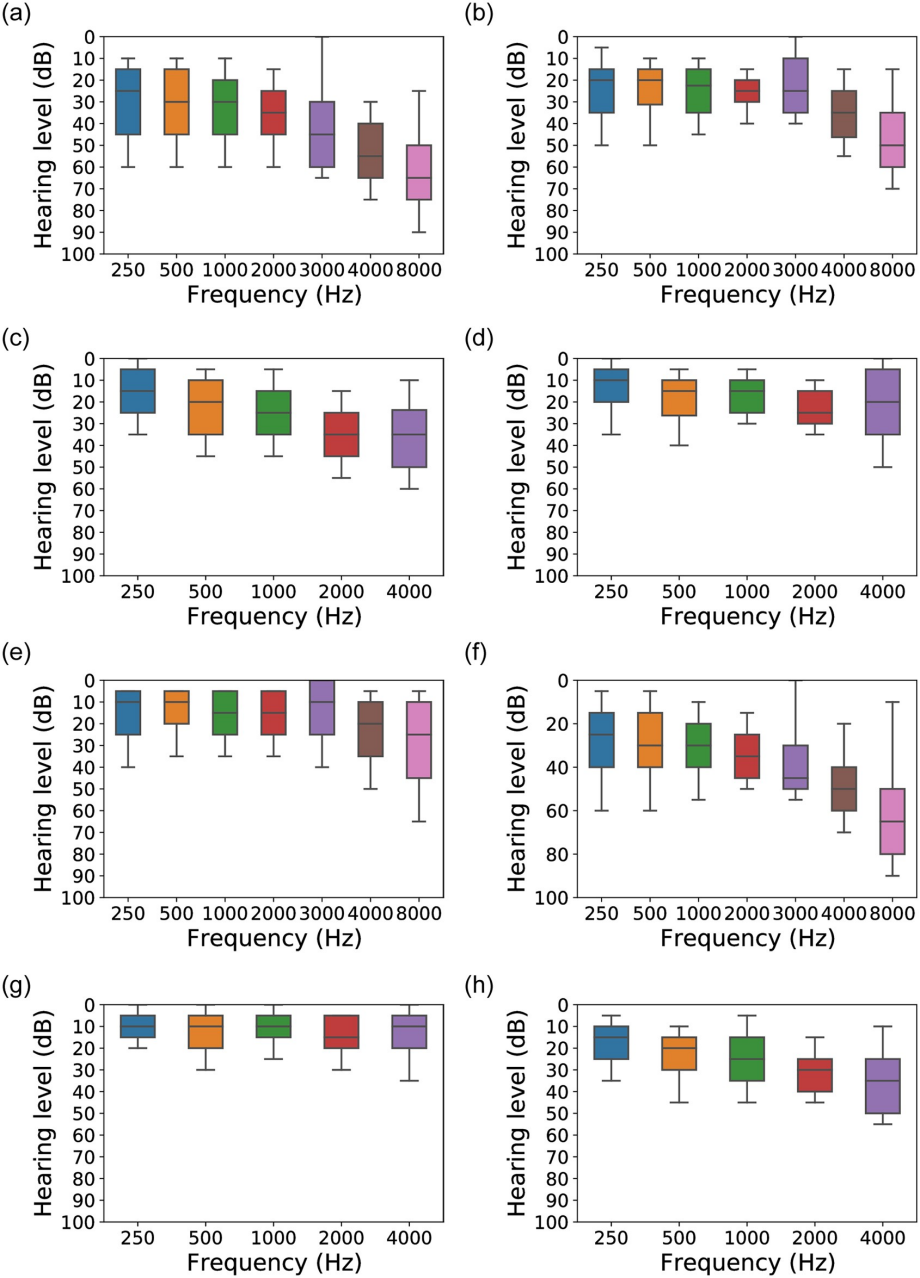
An average of pure tone audiograms (PTA) in all subjects’ AC and BC thresholds from 80-100 as shown in Fig 3. (a) True positive on the left AC SDS. The ‘80-89’ SDS is **correctly** classified as ‘80-89’. (b) False negative on the left AC SDS. The ‘80-89’ SDS is **misclassified** as ‘90-100’. (c) True positive on the left BC SDS. The ‘80-89’ SDS is **correctly** classified as ‘80-89’. (d) False negative on the left BC SDS. The ‘80-89’ SDS is **misclassified** as ‘90-100’. (e) True positive on the left AC SDS. The ‘90-100’ SDS is **correctly** classified as ‘90-100’. (f) False negative on the left AC SDS. The ‘90-100’ SDS is **misclassified** as ‘80-89’. (g) True positive on the left BC SDS. The ‘90-100’ SDS is **correctly** classified as ‘90-100’. (h) False negative on the left BC SDS. The ‘90-100’ SDS is **misclassified** as ‘80-89’.

To validate and better understand this, in Fig 5 we present the PTA threshold distribution for true positive (i.e., correctly classified) and false negative (i.e., misclassified) cases with varying AC/BC frequencies from our dataset. Specifically, Fig 5(a), 5(c), 5(e) and 5(g) show the true positive cases and the others present the false negatives. Here, we can visually notice that the “slope” of how the hearing level drops with increasing frequency is different for the two classes of plots (i.e., true positive sets vs. false negative sets). For example, compare the descending slope patterns for Fig 5(a) with Fig 5(b). Since the two sets of cases show significantly different patterns, it is no surprise that these samples could not be classified in the same category from a supervised machine learning model. What is more surprising is the similarity in slope patterns for the Fig 5(a) and 5(f) pair, Fig 5(b) and 5(e) pair, Fig 5(c) and 5(h) pair, and Fig 5(d) and 5(g) pair. Due to this similarity, a supervised machine learning model can misjudge that these two sample cases belong together. This unexpected similarity explains, on a data perspective, why our Random Forest model resulted in misclassifications for this data.

On a clinical perspective, the performance difference between the left and right ears is not a strange phenomenon. In previous auditory perceptual and physiological studies, this functional bias was reported, and the “right ear advantage” can be considered common for the human auditory system [41]. Specifically, auditory stimuli, which initiates from the right ear, passes through the cochlea to the cochlear nucleus and then ascends along both sides of the medulla oblongata. The cochlear nucleus on right side delivers about 70-90% of the total stimuli to the left superior olivary complex, and 10-30% of the stimuli goes to right superior olivary complex, which then ascends to the brain. As well-known, the left hemisphere of the brain is related to speech functions, and furthermore, the left primary auditory cortex has a preferential role in the temporal aspect of auditory stimuli [42, 43]. Thus, we can clinically hypothesize the left/right imbalance using the fact that the sound stimuli coming through right ear is more advantageous for the auditory functions. In this context, there have been several reports that the right ear showed better results in auditory rehabilitation and temporal resolutions [44–46]. Based on such observations, we can conjecture the that the Random Forest model’s performance (including its misclassifications) is a result of such auditory system characteristics.

### 4.3 Examining model performance without significant data imbalance

From our collected dataset, we noticed that the samples in the ‘90-100’ bin was over 60% of the entire dataset. Such phenomena can be common as this portion of the dataset represents normal cases. While we use an oversampling approach to account for the data imbalance over different bins, we wanted to make sure that this oversampling approach was effective even when reducing the level of data imbalance from the original dataset. For this, we tried removing the data included in the 90-100 bin from the dataset to train and test with only the samples included in the first 9 (of the original 10) bins (i.e., scores 0–89). As a result, we removed 7737 samples for the left ear and 7743 samples for the right ear from the original dataset for this experiment. Other parameters were kept consistent with the experiments in Section 3.3.

**Fig 6:**
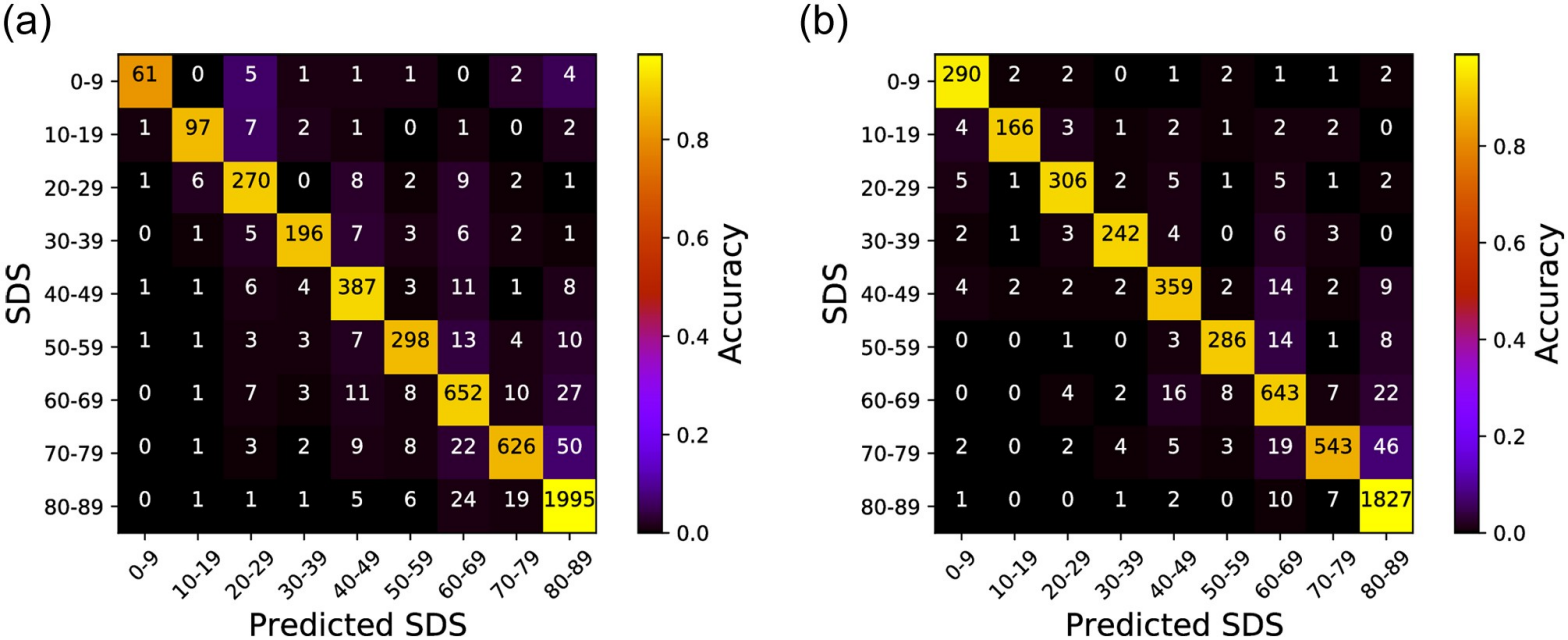
Confusion matrix for the SDS prediction performance with a range of 0-90 only. The X-axis shows the predicted SDS and the Y-axis shows the ground-truth. The numbers in the matrix present the number of prediction occurrences. An ideal confusion matrix would have bright colors (high occurrence counts) diagonally. (a) Left ear, (b) Right ear.

As the results in Fig 6 shows, we observed 92.72% and 94.10% accuracy in predicting SDS on the left and right ears, respectively. This suggests that our model shows good performance even when most of the normal-hearing listeners (i.e., from the 90-100 bin resulting in 60% of the entire dataset) are removed. This consistency in high prediction accuracy (with results in Section 3.3 where all data are included for accuracy measurements) suggests that our random forest model can be effectively and robustly trained using the oversampling approach mentioned in Section 2.1 despite the imbalance embedded in the data.

### 4.4 Clinical usage of the proposed model

We now discuss some examples of how PTA-SDS prediction can be used in clinical practice. It is clinically well known that patients with retrocochlear lesion experience loss in speech discrimination [47]. Thus, exceptional differences between the predicted and actual SDS can in fact be clinically useful information. Past clinical events such as head trauma or noise exposure as presented in Table 6 are closely related to the central auditory function performance [48, 49]. If the actual SDS is exceptionally high or low compared to the predicted SDS, and there is a low possibility of patient-induced malingering (or the patient can be associated with relevant clinically meaningful events), a more in-depth evaluation of the central auditory function may be recommended.

Such diseases showing relatively lower SDS compared to the pure-tone thresholds are grouped as Auditory Neuropathy Spectrum Disorder (ANSD). The cochlea of a patient with ANSD can detect sound stimuli; however, fails to send acoustic-generated signals to the brain [50]. Most patients with ANSD are diagnosed when they are too young to perform proper PTA and SDS tests. However, for some ANSD patients, hearing loss may progress slowly compared to the patients diagnosed at an early age, but faster than the normal population. From additional patient information such as Table 6, a physician can infer that patients with progressive hearing loss of unknown causes may be an effect of ANSD.

## 5 Conclusion

In this work, we examined the possibility of applying machine learning approaches for PTA score-based SDS prediction. Using PTA and SDS data collected from 12,697 subjects, we evaluated the performance of three different machine learning models as potential solutions. While the SVM and MLP models showed similar performances with pre-reported statistical model-based approaches, a Random Forest-based machine learning model was able to achieve high accuracy of more than 95% in identifying clinically-meaningful SDS from PTA thresholds inputs. Such systems can be applied directly to clinical practice given that their outputs can assist in more effectively (and easily) identifying patients needing detailed examinations.

## References

[1] Flint P., Haughey B., Robbins K., Thomas J., Niparko J., Lund V. et al. Cummings otolaryngology-head and neck surgery e-book. (Elsevier Health Sciences,2014)

[2] Lidén G. The scope and application of current audiometric tests. *The Journal Of Laryngology & Otology*. 83, 507–520 (1969) doi: 10.1017/S0022215100070651 5785649

[3] Dillon H. Hearing aids. (Hodder Arnold,2008)

[4] Hodgson W. Hearing aid assessment and use in audiologic habilitation. (Williams & Wilkins,1986)

[5] Mullins C. & Bangs J. Relationships between speech discrimination and other audiometric data. *Acta Oto-laryngologica*. 47, 149–157 (1957) doi: 10.3109/00016485709130328 13457874

[6] Pauler M., Schuknecht H. & Thornton A. Correlative studies of cochlear neuronal loss with speech discrimination and pure-tone thresholds. *Archives Of Oto-rhino-laryngology*. 243, 200–206 (1986) doi: 10.1007/BF00470622 3753299

[7] Fletcher H. A method of calculating hearing loss for speech from an audiogram. *Acta Oto-Laryngologica*. 38, 26–37 (1950) doi: 10.3109/00016485009127735 14818775

[8] Dijk J. Acoustic neuroma: Deterioration of speech discrimination related to thresholds in pure-tone audiometry. *Acta Oto-laryngologica*. 120, 627–632 (2000) doi: 10.1080/000164800750000450 11039874

[9] Yoshioka P. & Thornton A. Predicting speech discrimination from the audiometric thresholds. *Journal Of Speech*, *Language*, *And Hearing Research*. 23, 814–827 (1980) doi: 10.1044/jshr.2304.814 7442214

[10] Marshall L. & Bacon S. Prediction of speech discrimination scores from audiometric data. *Ear And Hearing*. 2, 148–155 (1981) doi: 10.1097/00003446-198107000-00003 7319153

[11] Cruz J. & Wishart D. Applications of machine learning in cancer prediction and prognosis. *Cancer Informatics*. 2 pp. 117693510600200030 (2006) doi: 10.1177/117693510600200030 PMC2675494 19458758

[12] Crowson M., Ranisau J., Eskander A., Babier A., Xu B., Kahmke R., et al. A contemporary review of machine learning in otolaryngology–head and neck surgery. *The Laryngoscope*. 130, 45–51 (2020) doi: 10.1002/lary.27850 30706465

[13] Tan A. & Gilbert D. Ensemble machine learning on gene expression data for cancer classification. *Proceedings Of New Zealand Bioinformatics Conference*. (2003) 15130820

[14] Kim H., Choi S., Ahn J., Yu H., Min K., Hong C., et al. Kaleidoscopic fluorescent arrays for machine-learning-based point-of-care chemical sensing. *Sensors And Actuators B: Chemical*. 329 pp. 129248 (2021), doi: 10.1016/j.snb.2020.129248 33446959 PMC7802756

[15] Ahn J., Nguyen Loc H., Krishna Balan R., Lee Y. & Ko J. Finding Small-Bowel Lesions: Challenges in Endoscopy-Image-Based Learning Systems. *Computer*. 51, 68–76 (2018) doi: 10.1109/MC.2018.2381116

[16] Park J., Cho H., Balan R. & Ko J. HeartQuake: Accurate Low-Cost Non-Invasive ECG Monitoring Using Bed-Mounted Geophones. *Proc. ACM Interact. Mob. Wearable Ubiquitous Technol*. 4 (2020,9), doi: 10.1145/3411843

[17] Ahn J., Kim H., Kim E. & Ko J. VOCkit: A low-cost IoT sensing platform for volatile organic compound classification. *Ad Hoc Networks*. 113 pp. 102360 (2021), doi: 10.1016/j.adhoc.2020.102360

[18] Park H., Lee Y. & Ko J. Enabling Real-Time Sign Language Translation on Mobile Platforms with On-Board Depth Cameras. *Proc. ACM Interact. Mob. Wearable Ubiquitous Technol*. 5 (2021,6), doi: 10.1145/3463498

[19] Raffin M. & Thornton A. Confidence levels for differences between speech-discrimination scores. *Journal Of Speech*, *Language*, *And Hearing Research*. 23, 5–18 (1980) doi: 10.1044/jshr.2301.05 7442184

[20] Hong Seong Ah L. Test-Retest Reliability of Speech Discrimination Test Using the Monosyllabic Word Lists. *Korean J Audiol*. 6, 128–135 (2002), http://www.ejao.org/journal/view.php?number=406

[21] He H. & Ma Y. Imbalanced learning: foundations, algorithms, and applications. (John Wiley & Sons,2013)

[22] He H. & Garcia E. Learning from imbalanced data. *IEEE Transactions On Knowledge And Data Engineering*. 21, 1263–1284 (2009) doi: 10.1109/TKDE.2008.239

[23] Adhikari S., Thapa S. & Shah B. Oversampling based Classifiers for Categorization of Radar Returns from the Ionosphere. *2020 International Conference On Electronics And Sustainable Communication Systems (ICESC)*. pp. 975-978 (2020)

[24] Japkowicz N. & Stephen S. The class imbalance problem: A systematic study. *Intelligent Data Analysis*. 6, 429–449 (2002) doi: 10.3233/IDA-2002-6504

[25] Moturu S., Johnson W. & Liu H. Predicting future high-cost patients: A real-world risk modeling application. *2007 IEEE International Conference On Bioinformatics And Biomedicine (BIBM 2007)*. pp. 202-208 (2007)

[26] Breiman L. Random forests. *Machine Learning*. 45, 5–32 (2001)

[27] Yoon H., RA H., Basaran C., Son S., Park T. & Ko J. Fuzzy Bin-Based Classification for Detecting Children’s Presence with 3D Depth Cameras. *ACM Trans. Sen. Netw*. 13 (2017), doi: 10.1145/3079764

[28] Park J., Nam W., Choi J., Kim T., Yoon D., Lee S., et al. Glasses for the Third Eye: Improving the Quality of Clinical Data Analysis with Motion Sensor-Based Data Filtering. *Proceedings Of The 15th ACM Conference On Embedded Network Sensor Systems*. (2017), 10.1145/3131672.3131690

[29] Pal S. & Mitra S. Multilayer perceptron, fuzzy sets, classifiaction. *IEEE Transaction On Neural Networks*. (1992) doi: 10.1109/72.159058 18276468

[30] Tauler R. & Walczak B. Comprehensive chemometrics: chemical and biochemical data analysis. (Elsevier,2009)

[31] Gislason P., Benediktsson J. & Sveinsson J. Random forests for land cover classification. *Pattern Recognition Letters*. 27, 294–300 (2006) doi: 10.1016/j.patrec.2005.08.011

[32] Guan H., Li J., Chapman M., Deng F., Ji Z. & Yang X. Integration of orthoimagery and lidar data for object-based urban thematic mapping using random forests. *International Journal Of Remote Sensing*. 34, 5166–5186 (2013) doi: 10.1080/01431161.2013.788261

[33] Kim H., Lee S., Min J., Kim E., Choi J., Ko J. et al. Fluorescent sensor array for high-precision pH classification with machine learning-supported mobile devices. *Dyes And Pigments*. 193 pp. 109492 (2021), doi: 10.1016/j.dyepig.2021.109492

[34] Menze B., Kelm B., Masuch R., Himmelreich U., Bachert P., Petrich W. et al. A comparison of random forest and its Gini importance with standard chemometric methods for the feature selection and classification of spectral data. *BMC Bioinformatics*. 10, 213 (2009) doi: 10.1186/1471-2105-10-213 19591666 PMC2724423

[35] Archer K. & Kimes R. Empirical characterization of random forest variable importance measures. *Computational Statistics & Data Analysis*. 52, 2249–2260 (2008) doi: 10.1016/j.csda.2007.08.015

[36] Pedregosa F., Varoquaux G., Gramfort A., Michel V., Thirion B., Grisel O., et al. Scikit-learn: Machine Learning in Python. *Journal Of Machine Learning Research*. 12 pp. 2825–2830 (2011)

[37] Fernández-Delgado M., Cernadas E., Barro S. & Amorim D. Do we need hundreds of classifiers to solve real world classification problems?. *The Journal Of Machine Learning Research*. 15, 3133–3181 (2014)

[38] Wilcoxon F. Individual comparisons by ranking methods. *Breakthroughs In Statistics*. pp. 196–202 (1992) doi: 10.1007/978-1-4612-4380-9_16

[39] Royston P. Approximating the Shapiro-Wilk W-test for non-normality. *Statistics And Computing*. 2, 117–119 (1992) doi: 10.1007/BF01891203

[40] Levene H. Robust tests for equality of variances. *Contributions To Probability And Statistics. Essays In Honor Of Harold Hotelling*. pp. 279-292 (1961)

[41] Tervaniemi M. & Hugdahl K. Lateralization of auditory-cortex functions. *Brain Research Reviews*. 43, 231–246 (2003) doi: 10.1016/j.brainresrev.2003.08.004 14629926

[42] Zatorre R. & Belin P. Spectral and temporal processing in human auditory cortex. *Cerebral Cortex*. 11, 946–953 (2001) doi: 10.1093/cercor/11.10.946 11549617

[43] Penhune V., Zatorre R., MacDonald J. & Evans A. Interhemispheric anatomical differences in human primary auditory cortex: probabilistic mapping and volume measurement from magnetic resonance scans. *Cerebral Cortex*. 6, 661–672 (1996) doi: 10.1093/cercor/6.5.661 8921202

[44] Sulakhe N., Elias L. & Lejbak L. Hemispheric asymmetries for gap detection depend on noise type. *Brain And Cognition*. 53, 372–375 (2003) doi: 10.1016/S0278-2626(03)00146-5 14607184

[45] Brown S. & Nicholls M. Hemispheric asymmetries for the temporal resolution of brief auditory stimuli. *Perception & Psychophysics*. 59, 442–447 (1997) doi: 10.3758/BF03211910 9136273

[46] Mondelli M., Santos M. & Feniman M. Unilateral hearing loss: benefit of amplification in sound localization, temporal ordering and resolution. *CoDAS*. 32 (2020) doi: 10.1590/2317-1782/20192018202 31721925

[47] Shibata T., Sakashita T., Yamane H. & Hashimoto C. Temporal resolution and speech recognition ability of patients with retrocochlear auditory dysfunction. *Acta Oto-Laryngologica*. 124, 30–34 (2004) doi: 10.1080/03655230410018417 15513507

[48] Bressler S., Goldberg H. & Shinn-Cunningham B. Sensory coding and cognitive processing of sound in Veterans with blast exposure. *Hearing Research*. 349 pp. 98–110 (2017) doi: 10.1016/j.heares.2016.10.018 27815131 PMC5645017

[49] Gallun F., Lewis M., Folmer R., Diedesch A., Kubli L., McDermott D., et al. Implications of blast exposure for central auditory function: a review. *Journal Of Rehabilitation Research & Development*. 49 (2012) 23341279 10.1682/jrrd.2010.09.0166

[50] Kaga K. Auditory nerve disease and auditory neuropathy spectrum disorders. *Auris Nasus Larynx*. 43, 10–20 (2016) doi: 10.1016/j.anl.2015.06.008 26209259

